# Lunar Regolith Improvement by Inducing Interparticle Adhesion with Capillary Forces

**DOI:** 10.3390/ma18102390

**Published:** 2025-05-20

**Authors:** Karol Brzeziński, Joanna Julia Sokołowska, Bartłomiej Przybyszewski

**Affiliations:** 1 Department of Roads and Bridges, Faculty of Civil Engineering, Warsaw University of Technology, 00-637 Warsaw, Poland; karol.brzezinski@pw.edu.pl; 2 Department of Building Materials Engineering, Faculty of Civil Engineering, Warsaw University of Technology, 00-637 Warsaw, Poland; 3 Faculty of Materials Science and Engineering, Warsaw University of Technology, Wołoska 141, 02-507 Warsaw, Poland; bartlomiej.przybyszewski@pw.edu.pl

**Keywords:** lunar soil, lunar geotechnics, lunar regolith simulant, LRS, extraterrestrial building materials, lunar base construction, lunar habitat construction

## Abstract

This paper concerns the assessment of the lunar regolith ability to consolidate in the presence of liquid water and develop and sustain cohesion after drying. This type of cohesion originates from interparticle adhesion and can be potentially improved through grading modification. The research was conducted using the lunar regolith simulant (EAC-1A) reproducing the PSD of real lunar soil delivered from the Moon. LRS was subjected to water and elevated temperature (equal to the highest temperature on the Moon) to produce specimens of consolidated material, CCR (Capillary-Consolidated Regolith) and to test flexural strength. In order to adapt to potentially small stresses, tests were performed according to the modified EN 196-1 procedure intended for Portland cement testing: specimens scaled to 20 mm × 20 mm × 80 mm (new molds with Polytetrafluoroethylene/Teflon^®^ coatings reducing adhesion were created), supports spacing in the three-point flexural test reduced to 50 mm and apparatus adjusted to precisely apply small loads. CCR developed flexural strength exceeding 0.025 MPa. Then, analogous tests were performed using LRS subjected to grinding in a disc mill prior to consolidation. It was shown that simple mechanical grinding enabled the improvement of interparticle adhesion with capillary forces, resulting in improved flexural strength of the consolidated material (0.123 MPa).

## 1. Introduction

After more than half a century since the first successful attempts at lunar exploration, which took place in the 1960s and 1970s, the time has come to resume this extraordinary project—in other words, mankind is on the threshold of the second phase of exploration of the Moon, the Earth’s natural satellite. The National Aeronautics and Space Administration (NASA) announced in 2020 that it planned to return to the Moon in 2024 and signed with Canada, Australia, Italy and Japan the so-called Artemis Accords [1] to jointly create an advanced and extensive international space exploration program, including Moon exploration. It is difficult to imagine large-scale lunar exploration without the need to create a stationary base for people. A place that—unlike space vehicles that quickly take passengers into orbit and deliver them to the surface of the Moon—should provide people with conditions sufficient not only for short-term, but also long-term stay. Such a place, which is also called a “lunar habitat”, should provide people with conditions proper for functioning and working without excessively endangering their safety and health.

Building a lunar habitat seems to be an extremely ambitious engineering and technological project that requires huge financial outlays. The cost of such a project may depend on many factors, including the applied technology, construction and material solutions, project scale, implementation time frame and financing strategy. When considering the choice of technology, the “astronomical” cost of transporting cargo from Earth to the Moon is always indicated, and although this cost is gradually being reduced with the development of space vehicle technology, it is still very high and calculated in the tens of thousands of dollars. In 2021, the cost of delivering 25 metric tons of cargo to the Moon using NASA’s heavy-lifting vehicle Space Launch System was estimated to be over USD 2 billion, which corresponded to ca. USD 75,000/kg of delivery cost [2,3,4]. The latest reports say that NASA has selected Intuitive Machines for the mission to deliver cargo to the Moon’s much more difficult to access South Pole area, where the lunar water (ice) was indicated [5,6]. The flight is scheduled for 2027 (operated by the Nova-C spacecraft) and it is estimated that delivering 79 kg of cargo will cost USD 116.9 million, which corresponded to ca. USD 1.5 million per kilogram [7].

**Figure 1:**
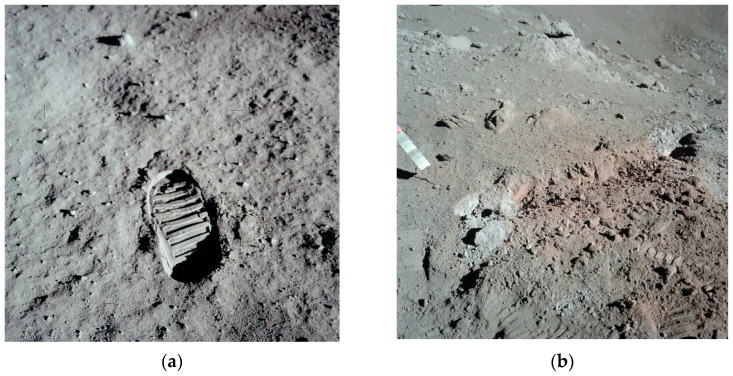
Photographs showing the lunar surface and compaction caused by the weight of a man taken during manned missions: (**a**) Apollo 11 mission in 1969—NASA Photo ID: AS11-40-5877; (**b**) Apollo 17 mission in 1972—NASA Photo ID: AS17-137-20986 (public domain, created by NASA).

The scope of the latest research on the construction of an extensive lunar habitat indicates that scientists are increasingly opting for solutions that maximize the use of resources available locally on the Moon, with minimal use of raw materials supplied from Earth. For this purpose, new building materials and concepts for modifying locally obtained lunar materials (such as lunar regolith) are being investigated and new concepts are being proposed and developed. These materials are generally referred to as “extraterrestrial materials” or “extraterrestrial composites”, and those that conceptually resemble concrete-like composites (phase of fillers combined or suspended in the matrix phase) are sometimes referred to as “lunar concrete” [8]. Attempts have been made to design and create composites with both sulfuric binders (liquefying and hardening as a result of temperature changes) [9] and polymer binders (e.g., from thermosetting resins or UV-cured) [8,10,11], and in the perspective of the possibility of obtaining lunar water, also hydraulic binders, such as Ordinary Portland Cement (OPC) [8,12] or geopolymers [11,13] or alkali-activation [14]. In the case of OPC, the most promising seems to be 3D printing technology, where the concrete mix acts as a filament [11,15]. In most cases, locally obtained fine-grained lunar regolith was considered the composite filler (aggregate). The chemical and granulometric characteristics of the filler were assumed based on the characteristics of real lunar regolith samples collected on the Moon during particular Apollo missions—astronauts have collected and delivered to Earth a total of 382 kg (842 pounds) of rock and lunar dust [16]. Figure 1 presents the surface of the Moon (outer layer of the regolith is clearly dusted) photographed by the astronauts in 1960s and 1970s.

In order to conduct tests of composites similar in material to the “designed” lunar composites, several researchers used so-called lunar soil/regolith analogues or lunar regolith simulant (LRS) developed on the basis of the real lunar regolith characteristics [17,18]. Theoretically LRS can be any material made from natural or synthetic earth or meteoric components to replicate one or more of the physical or chemical properties of the lunar regolith. It is, however, impossible to reproduce an exact lunar soil, i.e., material that would reflect all physical, mechanical, electromagnetic and thermal properties, as well as the chemical or mineralogical composition using only rocks from Earth. For this reason, all analogues of lunar regolith are produced to simulate one or two of the aforementioned characteristics necessary for research and experimentation. Moreover, all LRS are produced by mechanical rock crushing in order to better reflect the particle size distribution and shape of the particles; the rocks are crushed by impacts.

The authors attempted before to use the lunar regolith analogue marked AGK-2010 that mapped the grain size distribution (of uniformity coefficient, Cu = 3.54 [17]) developed by the Space Research Center of the Polish Academy of Sciences. It was used to produce a “lunar mortar” with a reduced water content (low water/cement ratio) and a high content of superplasticizer, optimized to obtain the proper rheological properties (consistency and plasticity) of fresh mortar important in the context of 3D printing technology [12]. The research results were promising—the possibility of using lunar regolith as an aggregate in concrete-like composites with a cement binder was confirmed. Nevertheless, the amount of water necessary for proper hydration of cement binder—although significantly reduced from the point of view of concrete technology—still seemed to be too high in the context of its very low availability on the Moon.

In the research presented in this paper, a different approach to the production of a building composite was applied—water was used not as a component that is incorporated into the hydrated phase of the mineral binder, but as a technological agent—recoverable and ready for reuse, i.e., functioning in a closed cycle. The “bond” between regolith particles occurred not as a result of a chemical but physical process of capillary consolidation; thus, the resulting material is characterized by the same composition as before consolidation, but with a stronger structure. In the research again LRS was used, but this time it was the lunar regolith analogue marked EAC-1A developed in the European Astronaut Centre for the needs of advanced research and validation conducted in the European Lunar Exploration Laboratory (LUNA) [19]. It was intended to reproduce the material from a dusty outer layer of lunar regolith that was subjected to further processing and testing.

The method proposed by the authors is intended to improve the mechanical properties of lunar regolith without the need for importing materials from outside the Moon (except for the initial startup requirements such as machinery and possibly water, although, as mentioned above, the latter can also be found “on-site”). The method capitalizes on the natural tendency of particles to adhere [20], a characteristic also exhibited by regolith grains. However, the Moon lacks a crucial factor that could stimulate this tendency—liquid water. Seiphoori and colleagues demonstrated that even among relatively large particles (approximately 5 μm), a lasting coherence can be induced through the transient action of capillary forces [21]. During the drying process of a mix of fine particles and water, two significant phenomena occur. Firstly, capillary forces help overcome electrostatic repulsion forces between grains, allowing for atomic contact and thereby promoting adhesion/cohesion. Secondly, fine particles are drawn to the points of contact between larger particles (forming solid bridges), further stabilizing the connection. By wetting and drying the regolith, coherence can be induced, which remains durable even after complete drying. This approach exploits the inherent potential of the material without the need for additional components. Thus, authors proposed a method of transforming loose lunar dust into a solid material by capillary consolidation (CCR—Capillary-Consolidated Regolith). Many researchers investigated how air-drying, or oven-drying of soil, affects its engineering properties, primarily focusing on changes in plastic properties and the gradation of cohesive soils due to particle aggregation [22,23,24]. Typically, methodologies involve testing the material after or during rewetting [25,26,27]. Nevertheless, there are also studies examining the strength of dry or near-dry clays [28,29]. The obtained strength is relatively modest, but considering the lower gravity on the Moon, various applications for such building material are conceivable (substructures, insulation barriers, etc.). Although rewetting may lead to degradation of material properties, the risk of such an occurrence in space is negligible. In the framework of our research, specimens of the CCR made of EAC-1A lunar regolith simulant suitable for testing the flexural strength in a three-point bending test were prepared. Next, the origins of such material’s strength and proposed further improvement by prior milling of the regolith (in the disc mill) that significantly improved performance of the material were discussed. Finally, the strength of the consolidated ground material was compared with the strength of the “original” LRS and the implemented approach in the light of other improvement methods, and its potential applications were discussed.

## 2. Materials and Methods

### 2.1. Theoretical Foundations of Capillary Consolidation

The proposed concept of capillary consolidation utilizes the mechanism described by Seiphoori and colleagues [21]. Initially, particles suspended in water repel each other due to electrostatic forces. However, as the water evaporates, capillary bridges form between the particles, inducing temporary apparent cohesion that disappears upon complete drying. The drying phase itself is critical: as the capillary bridges shrink and the attractive forces intensify, drawing particles closer together. If these capillary forces become sufficiently strong, they overcome electrostatic repulsion, causing the particles to come into direct molecular contact. At this stage, cohesion arises primarily from van der Waals interactions, which are relatively weak compared to other cohesion mechanisms, such as chemical bonding, electrostatic attraction or capillary forces. Nevertheless, when particles are sufficiently small, these interactions can create stable aggregates capable of resisting various loads (e.g., rewetting [21] or mechanical loading, as demonstrated in our work).

Numerous factors influence this phenomenon at micro- and nano-scale levels, including the surface tension of the liquid, particle surface potential, drying temperature, and particle shape. Seiphoori et al. [21] studied and explained this mechanism at the particle level using relatively simple particle geometry and composition (amorphous silica spheres). While their theory serves as a guiding principle for our research, the inherent complexity of the materials involved leads us to concentrate on macroscopic observations and measurements with direct practical implications.

### 2.2. Material

**Figure 2:**
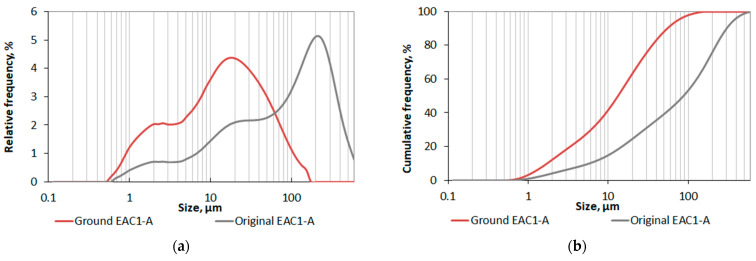
Particle size distribution of the tested EAC-1A lunar regolith simulant in the original and milled (ground) state: (**a**) relative frequency plot; (**b**) cumulative frequency plot.

EAC-1A lunar regolith simulantis a basanite material quarried from Siebengebirge Volcanic Field, Königswinter, Germany. Due to the high demand for regolith simulant in LUNA’s experiments, the material was selected to reasonably reproduce properties of lunar regolith at a relatively low cost. Compared to the material obtained by the Apollo-17 mission, the EAC1-A is similar with regard to sphericity and particle size distribution (PSD). Figure 2 shows PSD plots of the original material and its modified ground version (both were used in our experiment). The PSD measurements were performed by the laser scattering method using the laser analyzer Horiba LA-300 (Kyoto, Japan). The test involved passing laser beams through a 0.1% sodium hexametaphosphate (CAS: 1012-56-8) solution containing particles of tested material dispersed by ultrasonics and determining the particle size (in the range of 0.01–600 μm). The obtained values of statistical parameters describing both PSD as well as specific surface area (calculated from PSD, making an assumption about the spherical shape of the particles) are given in Table 1.

Moreover, in the case of the tested EAC-1A lunar regolith simulant, it’s major elementalcomposition is relatively close to the one of the original lunar regolith samples. Nevertheless, EAC-1A contains more alkalis (4.20% of Na_2_O + K_2_O, by wt.) compared to lunar materials (0.40–1.32%, by wt.; full description can be found in [19]). According to concrete technology, the high content of alkalis is not desirable because they may lead to alkali-aggregate reactions, including an alkali–silica reaction (ASR) or alkali–carbonate reaction (ACR) between particular aggregates (e.g., opal, chalcedony, cryptocrystalline quartz and sodium and potassium hydroxides) [30]. As a result, swelling products are formed and in extreme cases may lead to damage of the microstructure and also, consequently, the macrostructure of the composite. In the presented research, the authors do not analyze such a case, however, one should have in mind the potential reaction caused by alkalis in terms of other extraterrestrial building materials made of lunar regolith.

As for the basic physical properties of the used lunar regolith simulant, worth mentioning are the bulk density of 1450 kg/m^3^, absolute density of 2900 kg/m^3^, and the cohesion of the compacted material equal to 0.38 kPa (at a bulk density of 1950 kg/m^3^) [19].

### 2.3. Specimens’ Prepration

**Figure 3:**
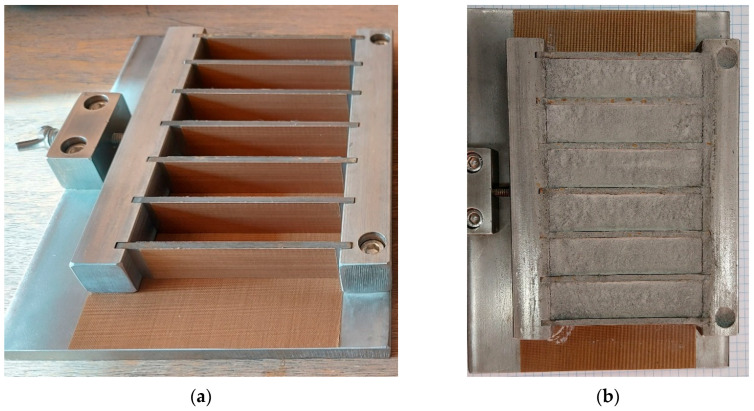
Specimens’ preparation: (**a**) PTFE-coated custom-made steel mold; (**b**) capillary consolidated LRS specimens after drying procedure.

The major property analyzed in the presented research is the mechanical strength developed during capillary consolidation of the material. The proposed testing procedure was elaborated by adopting the approach described in the EN 196-1:2016 methods of testing cement and determination of strength [31], with some modifications. All the dimensions of the prism-shaped specimens were reduced by a factor of 2.0; yet, the aspect ratio was preserved. Hence, the tested specimens had approximate dimensions of 20 mm × 20 mm × 80 mm. First of all, the reduced size facilitated the handling of the specimens. Due to the scale effect and lower mass to strength ratio, smaller objects are less likely to break during transport, etc. Furthermore, reducing the linear size of the sample by a factor of 2.0 decreased the amount of the material required for one sample by a factor of 8.0. It is beneficial when the availability of materials is low. Moreover, the used material was finer than specified in the standard test [31]. Hence, even with downsized molds, a high (>40) minimum specimen dimension to grain size ratio was preserved. The specimens were molded in custom-made steel molds coated with Polytetrafluoroethylene (PTFE) sheets, reducing the adhesion between the steel and tested material (see Figure 3a). The procedure workflow was as follows:

- Preparing fresh mix, by mixing 400 g of dry LRS material with 100 g of distilled water (water/LRS ratio of 0.25);
- Pouring the fresh mix into the molds;
- Air-drying in ambient conditions for 2 h to drain excess water and remove air bubbles from the mix;
- Oven-drying at a temperature of 127 °C for 22 h;
- Applying markers for digital image correlation (DIC) measurements.

**Figure 4:**
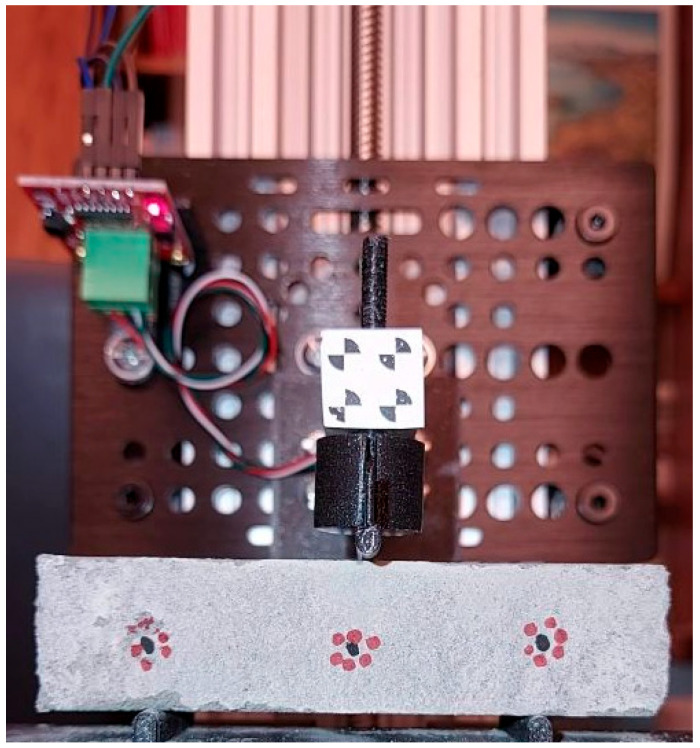
The flexural strength test performed on CCR prism specimen of size 20 mm × 20 mm × 80 mm in the three-point flexural test (the force concentrated in the middle of the 50 mm span); the markers were used for tracking the deflection.

One optical marker was applied in the middle of the specimen (for tracking deflection), and two more 25 mm to the side (for placing above the supports of the bending module and for reference scale—see Figure 4 in the next section).

### 2.4. Flexural Strength Test

After scaling the specimens’ size to 20 mm × 20 mm × 80 mm, it was also necessary to adjust the static system dimensions and loading rate: the flexural strength of the consolidated material, CCR was tested in a 3-point flexural test with support spacing of 50 mm (Figure 4), and the loading rate was set to 0.1 mm/s.

Due to the load cell’s low stiffness, the crosshead’s resultant velocity was smaller. Hence, the deflection was additionally measured using the digital image correlation technique by point tracking with the Lucas–Kanade optical flow algorithm [32] implemented in the OpenCV library [33]. Stress and strains were computed based on the measured force and deflection of the specimens using Equations (1) and (2), respectively:

(1) $$\sigma =\frac{3\times F\times L}{2\times B\times H^{2}},$$

(2) $$\epsilon =\frac{6\times d\times H}{L^{2}},$$

where

*σ* is the flexural stress (MPa);

*F* is the loading force (N);

*B* is the specimen width (mm);

*H* is the specimen height (mm);

*L* is the support spacing (mm);

*d* is the specimen deflection (mm);

*ϵ* is the flexural strain (-).

### 2.5. Microscopic Observations

The lunar regolith simulant microstructure was observed using Hitachi TM-3000 Scanning Electron Microscope (SEM) (Tokyo, Japan). Fractured specimens with the size of approx. 20 × 20 mm were acquired from manufactured specimens (remaining parts after bending test), glued to the microscope stage using carbon tape and transferred to the microscope chamber. Observations were carried out in SE mode using magnifications up to ×1000 with an accelerating voltage of 15 kV.

## 3. Results and Discussion

Taking into consideration the PSD plots (Figure 2) as well as data describing the distributions and specific surface area given in the following Table 1, it was confirmed that grinding the lunar regolith in the Fritsch PULVERISETTE 13 disc mill (Idar-Oberstein, Germany) had a positive effect on the grading from the point of view of potential consolidation. Firstly, both the minimal and maximal particle sizes recorded for the ground EAC1-A are lower than in the case of the original one, and although the minimal size of ground material is only 13.4% smaller, its maximal recorded particle size is 152.45 µm, which is almost four times lower than the initial EAC1-A’s minimal size. Secondly, the grain size distribution has also changed quantitatively, although the plot has retained a similar character.

The statistical parameters analyzed, i.e., the median, mean and mode of the ground material, adopted values lower by 84.8%, 82.4% and 91.3% (respectively) than the values of the original EAC1-A. That confirmed that the ground LRS is much finer in the entire range of distribution. During the PSD tests, based on the recorded particles’ sizes, the specific surface area of both analyzed materials was also determined. Milling the material allowed for obtaining a much more developed specific surface area (it was over 2.5 times larger than in the case of the original LRS). However, it should be remembered that the results of the last determination are calculated based on individual PSDs, assuming a spherical shape of the particles (thus estimated to some extent).

The PSD analysis of the original and ground EAC-1A lunar regolith simulants allowed us to conclude that the latter is more likely to capillary consolidate, which was later confirmed by the means of the mechanical test and microscopic observations.

**Figure 5:**
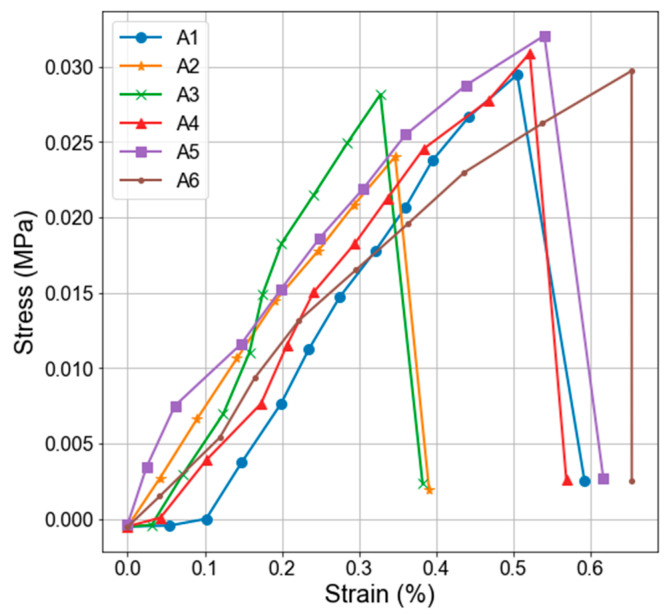
Flexural strength test results of EAC-1A lunar regolith simulant improved by capillary consolidation.

The lunar regolith in the original state (i.e., un-ground) due to the consolidation developed enough strength to be handled and tested in a three-point flexural test. Figure 5 presents the detailed results of the strength test. The specimens showed flexural strength exceeding 0.025 MPa (mean value of 0.029 MPa), which is relatively small compared to the typical structural materials.

**Figure 6:**
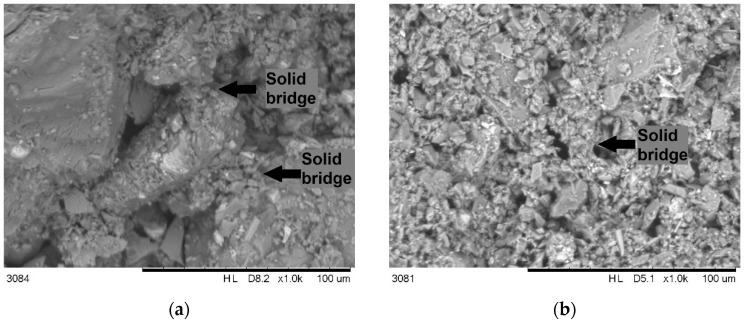
SEM micrographs of fractured samples of CCR: (**a**) original PSD; (**b**) ground material.

During capillary consolidation, two important phenomena occurred, the effects of which were positive on developing cohesion in the material [21]. Firstly, capillary forces allowed the repulsive barriers between grains to be overcome. Once the grains leaped into true contact, they formed a stable bond that persisted after completely drying the material (and the absence of capillary forces). Secondly, the smallest particles were dragged into the contact zones of larger ones. Such connections are called solid bridges. The presence of such bridges was observed in SEM images (Figure 6). Due to the size effect, the smaller particles turned out to be connected with relatively stronger bonds (due to the higher surface-to-mass ratio). Hence, the solid bridges stabilized contacts between the larger particles.

**Figure 7:**
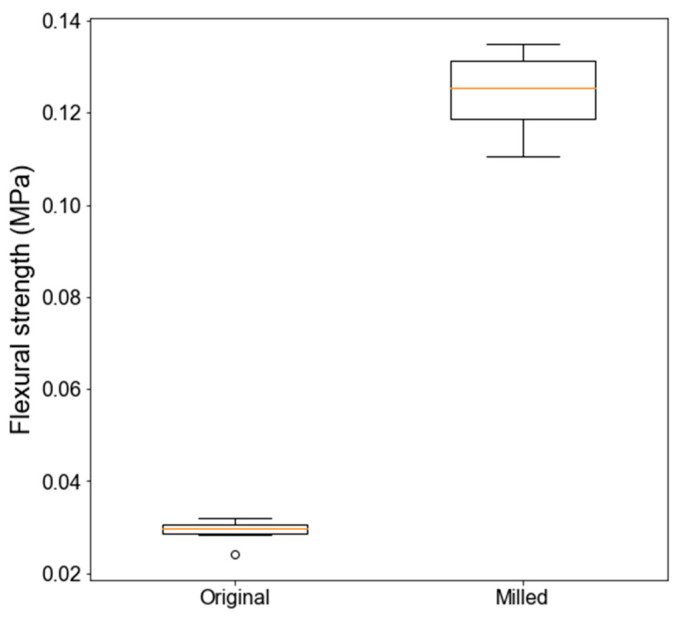
Comparison of flexural strength of CCR produced from original and ground LRS.

One can note that finer material may exploit the size effect even more. It can be achieved with a two-step process of lunar regolith improvement (without introducing any other material). First, the lunar regolith is milled (see the red line in Figure 2). In the second step, it is subject to capillary consolidation. In such a case, much stronger cohesion develops, resulting in approximately four times greater flexural strengths (mean strength of 0.123 MPa), as presented in Figure 7.

The experiment confirmed that capillary consolidation transformed loose dust into a solid material. The strength of such a material is relatively small, compared to other improvement techniques (see Table 2). Nevertheless, its handling is easier due to its structural soundness.

**Table 2:** Comparison of flexural strength of regolith simulants obtained with different binding techniques.

Improvement Technique	Flexural Strength (MPa)	Reference
Capillary Consolidation—original PSD	0.029	this study
Grinding + Capillary Consolidation	0.123	this study
Sintering	0.23–0.55	[34,35]
Portland cement binder	6.8	[12]
Polylactide binder	0.43–19.01	[36]
Alkali-activation	7.16–8.80	[14]

**Figure 8:**
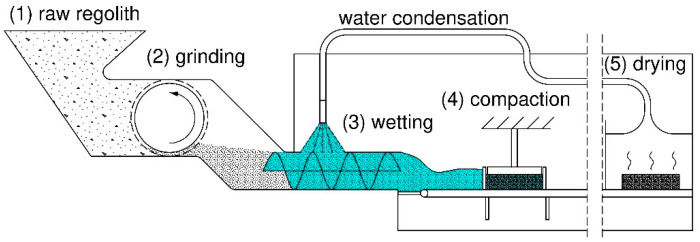
Schematic workflow of processing the lunar regolith by capillary consolidation and additional compaction phase.

Moreover, considering the Moon’s low gravity (six times smaller than on Earth, i.e., 1.62 m/s^2^ [37]), the potential applications of the improved regolith include building insulation barriers [38,39] or bottom layers of pavements and foundations. Besides the improved mechanical properties of the CCR, it is easier to handle because the smallest particles are bound in the material. In the case of unbound regolith, the loose lunar dust causes various problems [40]. Moreover, the properties of the material could be improved by other techniques, such as compaction before drying to improve the contact network between particles or preloading to increase the fatigue life of the material [41,42]. An example workflow of processing the lunar regolith by capillary consolidation and additional compaction phase is visualized in Figure 8.

In the presented conceptual workflow, the raw regolith is milled to obtain finer and more uniform material. The finer particles also result in greater capillary forces in the drying phase. Moreover, the larger specific surface area is developed, making the adhesive forces between particles more significant. It should be emphasized that even though the water is utilized in the process, its consumption will be minimal because the water will be recycled, with small amounts potentially being bound on the particles’ surface. The development of such technology requires further research and solving engineering problems (e.g., adapting existing recovery techniques such as conventional condensation or membrane condensers).

## 4. Conclusions

The aim of the study presented in this paper was to indicate that it is possible to stabilize the dusty outer layer of the lunar regolith without the need for additional stabilizing agents, but only in the process of capillary consolidation of the regolith. The tests conducted on a regolith simulant EAC-1A allow for the following conclusions:

- The action of capillary forces can induce interparticle adhesion in loose regolith simulant; this way, a solid material showing a minimal flexural strength of 0.029 MPa can be created;
- CCR produced with the finer material shows greater strength; grinding of the regolith simulant allowed to obtain material four times stronger (flexural strength of 0.123 MPa);
- An additional advantage of binding fine particles of lunar regolith technology is creating a locally cleaner (dust-free) environment, which is beneficial for the durability of the equipment used on the Moon, especially the optical parts;
- The proposed workflow does not require additional materials, and water utilized in the process can be reused;
- This approach could be developed in the future, applying methods of improving intrinsic material properties without additives (e.g., as compaction).

Although today, the use of water in such a process performed on the Moon might seem unjustified, in light of NASA’s plans to obtain ice water from the lunar poles, this approach gains credibility. The possibility of using lunar water completely changes the approach to producing extraterrestrial building materials and constructing the lunar base. Moreover, our proposed solution includes water recovery and its reuse in the subsequent cycles. It should be noted however that the results confirm that the capillary consolidation mechanism is functioning, without showing its actual limits. Finding an optimal solution will require systematic studying of the influence of many factors (e.g., water content, drying duration and temperature, various size distributions, compaction influence, etc.). Furthermore, depending on the specific application, more testing is required, especially in terms of structural performance (e.g., compressive strength).

## Figures and Tables

**Table 1:** Statistical parameters describing the particle size distribution of the tested EAC-1A lunar regolith simulant in the original (un-ground) and milled (ground) state.

Parameter	EAC-1A Lunar Regolith Simulant	
	Original	Ground
D_min_ [µm]	0.67	0.58
D_10_ [µm]	5.87	1.73
D_50_ (median) [µm]	86.36	13.14
D_m_ (mean) [µm]	123.53	21.70
Mode [µm]	187.33	16.25
D_max_ [µm]	592.39	152.45
SPA ^1^ [cm^2^/cm^3^]	4542.7	11,929.0

^1^ SPA, i.e., specific surface area was calculated from the distribution, making an assumption about the spherical shape of the particles.

## Data Availability

The original contributions presented in this study are included in the article. Further inquiries can be directed to the corresponding author.

## Abbreviations

The following abbreviations are used in this manuscript:

PSD	Particle size distribution
EAC-1A	European Astronaut Centre lunar regolith simulant 1
CCR	Capillary-Consolidated Regolith
EN	European Norm
LRS	Lunar regolith simulant
NASA	National Aeronautics and Space Administration
OPC	Ordinary Portland Cement
LUNA	Proper noun of European Lunar Exploration Laboratory
CAS	Chemical Abstracts Service
ASR	Alkali–silica reaction
ACR	Alkali–carbonate reaction
PTFE	Polytetrafluoroethylene
DIC	Digital image correlation
TM-3000	Table Microscope 3000—proper noun of the Hitachi microscope model
SEM	Scanning Electron Microscope
D_min_	Minimal diameter
D_10_	Diameter/size below which 10% of all particles are found
D_50_	Median diameter
D_m_	Mean diameter
D_max_	Maximal diameter
SPA	Specific surface area
